# Metaheuristic Algorithm-Based Vibration Response Model for a Gas Microturbine

**DOI:** 10.3390/s22124317

**Published:** 2022-06-07

**Authors:** L. A. Montoya-Santiyanes, Omar Rodríguez-Abreo, Eloy E. Rodríguez, Juvenal Rodríguez-Reséndiz

**Affiliations:** 1 Industrial Technologies Division, Universidad Politécnica de Querétaro, Carretera Estatal 420, El Marques 76240, Mexico; omar.rodriguez@upq.edu.mx; 2 Red de Investigación OAC Optimización, Automatización y Control, El Marques 76240, Mexico; 3 Centro de Ingeniería y Desarrollo Industrial (CIDESI), Querétaro 76125, Mexico; eloy.rodriguez@cidesi.edu.mx; 4 Facultad de Ingeniería, Universidad Autónoma de Querétaro, Querétaro 76010, Mexico; juvenal@uaq.edu.mx

**Keywords:** microturbine, vibration analysis, metaheuristic algorithm, response surface

## Abstract

Data acquisition and processing are areas of research in fault diagnosis in rotating machinery, where the rotor is a fundamental component that benefits from dynamic analysis. Several intelligent algorithms have been used to optimize investigations of this nature. However, the Jaya algorithm has only been applied in a few instances. In this study, measurements of the amplitude of vibration in the radial direction in a gas microturbine were analyzed using different rotational frequency and temperature levels. A response surface model was generated using a polynomial tuned by the Jaya metaheuristic algorithm applied to the averages of the measurements, and another on the whole sample, to determine the optimal operating conditions and the effects that temperature produces on vibrations. Several tests with different orders of the polynomial were carried out. The fifth-order polynomial performed better in terms of MSE. The response surfaces were presented fitting the measured points. The roots of the MSE, as a percentage, for the 8-point and 80-point fittings were 3.12% and 10.69%, respectively. The best operating conditions were found at low and high rotational frequencies and at a temperature of 300 ${}^{\circ }$C. High temperature conditions produced more variability in the measurements and caused the minimum value of the vibration amplitude to change in terms of rotational frequency. Where it is feasible to undertake experiments with minimal variations, the model that uses only the averages can be used. Future work will examine the use of different error functions which cannot be conveniently implemented in a common second-order model. The proposed method does not require in-depth mathematical analysis or high computational capabilities.

## 1. Introduction

Unbalanced forces, internal and external excitations, complex work environments, and other causes can trigger excessive vibration in rotating systems [1]. The rotor is one of the basic components evaluated in the dynamic analysis of machinery since its misalignment and imbalance can lead to bearing defects [2]. It is crucial to monitor the rotational frequency as the frequencies of vibration associated with the most commonly occurring faults, such as imbalance, misalignment, backlash, and bearing orbits, are multiples or percentages of this frequency [3].

Many theoretical, numerical, and experimental investigations aiming to determine optimal working conditions for rotary systems and to evaluate the influence of various factors have been undertaken. Temperature is one of the most important of these factors. The temperature differential between the turbine bearings and the compressor in a turbocharger rotor can be up to 50 ${}^{\circ }$C, which means that bearings operate under different conditions [4]. Giomiciaga and Keogh [5] studied the heat fluxes in a hydrodynamic bearing by decomposing the circular whirl orbits, and found that the surface temperature differentials in the journal bearing could cause significant rotor thermal bending. Zadorozhnaya E. et al. [6,7] used an orthogonal central composite model to estimate the thermal state of the bearings associated with the dynamics of a turbocharger rotor within a measurement range of 0–350 ${}^{\circ }$C. Gao P. et al. [8] studied the effect of imbalances in low and high-pressure rotors, considering the non-linear thermal behaviors of an inter-shaft bearing. These affected the behavior of the resonance zones, causing a shift in the frequencies observed. Gu and Chu [9] studied the dynamics of a rotor shaft with a constant cross-sectional area which was subjected to thermal impacts and determined the temperature distributions in the space and time domain. It was found that thermal vibration was influenced by several factors including the shaft length, rotational speed, heating position and critical speed. Ribeiro P. and Manoach E. [10] investigated the effects of variation in temperature, thickness, and radius of curvature on the dynamics of curved beams using a hierarchical finite element method (FEM). They determined that, with positive temperature variations, temperature and curvature produced forces that increased stiffness. Weipeng Sun et al. [11] determined that oil-whirl amplitude increased with rotational speed and that the temperature of the oil film had a high impact on the stability of a bearing rotor system. Sławiński D. et al. [12] analyzed causes of blade failure in a high-power gas turbine, focusing on thermal analysis since the failure occurred in the absence of symptoms from the casing. They determined that changes in the type of fuel caused temperature increases in the blade material, causing elongation and later abrasion against the turbine body. Other commonly reported phenomena in turbomachinery include the Morton effect (ME) which produces synchronous thermal instability and spiral vibrations of rotors when there is significant differential heating at rotor journal bearings [13]. Tong X. et al. [14] suggested that one of the main effects of shaft thermal expansion was the ME which resulted in thermally induced synchronous instability because of the asymmetric distribution of temperature. They considered the ME in a dynamic model of a shaft operating at different speeds and temperature differentials, and predicted a greater amplitude of vibration when the ME was included compared to a model that did not consider it. Plantegenet T. et al. [15] determined that the ME can be triggered by imbalances when the rotor operates near the first flexible mode—synchronous vibration amplitudes must be long enough for the heat to build up in the lubricant layer for the ME to occur.

Most of the previous research has considered the issues at a component level. However, there is little research that has focused on the characteristics of the fault or that has sought to introduce intelligent algorithms to examine structural rotor faults and their associated properties [16]. Metaheuristic algorithms perform well when applied to various engineering problems. For example, they have been applied to gear system design, cam design, wind turbine blade design, and to the design of aeronautical equipment and combustion systems [17]. Several techniques have been applied to handle vibrations using metaheuristic algorithms to increase efficiency by maximizing the amplitude of the defect frequency [18]. They have also been applied to minimize the peak accelerations in the structural analysis of a seismic isolated frame [19]. Fiori de Castro H. et al. [20] used the experimental responses of a rotary system and a metaheuristic hybrid algorithm to tune the magnitude of the unbalanced force, axial position, and phase. The advantage of using these algorithms is that they are less sensitive to noise in the search space, making them suitable for optimizing systems and finding solutions; they also tend to improve over generations.

Several studies have included the effect of temperature on structural dynamics as well as the use of intelligent algorithms, with the genetic algorithm (GA) being the most widely applied in mechanical design applications, such as gear trains, aerodynamic components and combustion equipment [21]. However, it is difficult to find optimization applications which have applied Jaya in mechanical systems, even though it is easy to implement and produces a low level of error. Moreover, available studies have not addressed variability in the experimental measurements, nor have they considered variability in the algorithm inputs. Therefore, this paper presents a response surface model for vibration amplitude in a gas microturbine using a high-order polynomial model. The methodology is described in Section 2. Section 2.1 details the experimental setup for obtaining the vibration data from the microturbine, considering the effect of rotational frequency and temperature at several levels. Section 2.2 focuses on a metaheuristic algorithm for tuning the coefficients of a polynomial model such that the mean square of the error (MSE) is minimized. After determining the best order, response surfaces were fitted using the average of each level (8-point) and later using all the levels and their replicas (80-point). In Section 3, the response surfaces fitted to the measurements are reported, where the roots of the MSE as a percentage, obtained using the 8-point and 80-point samples, were 3.12% and 10.69%, respectively. The study conclusions are described in Section 5, with discussion of the optimum operating conditions in terms of vibration amplitude and the influence of temperature on the system characteristics.

## 2. Methods

This section details the experimental procedure used to obtain the vibration data from the microturbine. Subsequently, the process of obtaining the response surface employing polynomials fitted by a metaheuristic algorithm is described.

### 2.1. Experimental Setup

**Figure 1:**
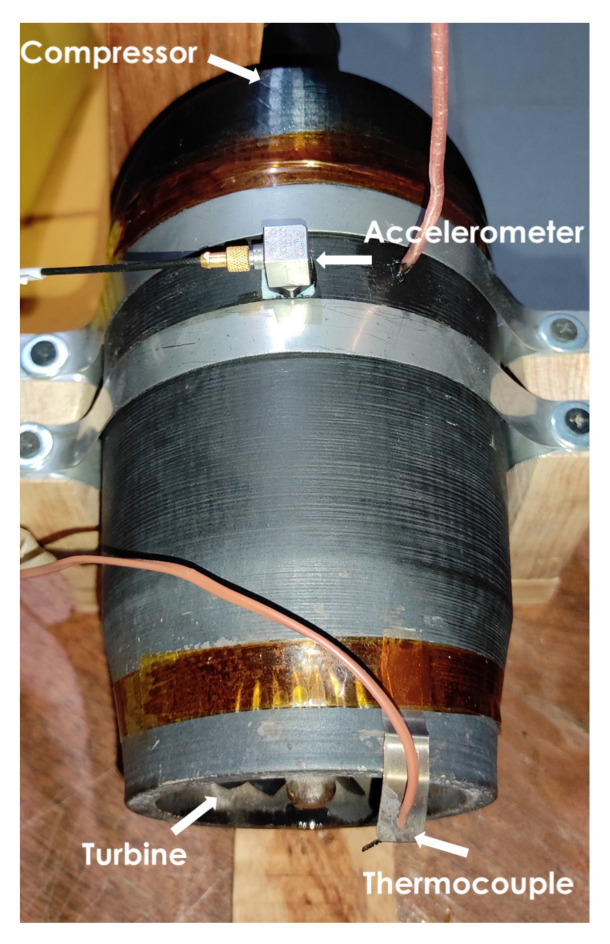
Microturbine.

A microturbine was used to carry out the experiments (see Figure 1), which was composed of a 3D-printed compressor with PLA, a 304 stainless steel turbine, and an HSS steel shaft. The fuel used consisted of butane/propane gas with a maximum working pressure of 3.5 kg/cm${}^{2}$. A piezoelectric accelerometer (PCB Piezotronics, Model: 333B30, Sensitivity: 98.2 mV/g) was used to measure the acceleration response. Table 1 summarizes the technical data for the microturbine used in the experiment. The data acquisition systems for the amplitude and temperature measurements consisted of the National Instruments NI-9234 and NI-9211 modules, respectively, and a cDAQ-9174 chassis. A sound and vibration toolkit from LabVIEW was used for signal processing. The accelerometer was positioned close to the bearing on the side of the compressor wheel (see Figure 1), avoiding contact with the support straps of the system.

Most rotor or shaft failures occur at speeds which coincide with the rotational frequency or multiples of it, including harmonics, or subharmonics [16]. A constant air feed source was used at the microturbine inlet. The maximum rotational frequency reached in this way was 127 Hz. Therefore, it was decided to use the nominal frequencies of 27, 76, and 127 Hz, equivalent to 1620, 4560, 7620 rpm, respectively, representing different levels of this factor. The frequencies used were similar to those used in Surajkumar Kumbhar et al. [22] although these authors mainly focused on bearings.

The microturbine was operated at three nominal temperature levels (i.e., 150, 300, and 500 ${}^{\circ }$C), measured at the outlet of the turbine stage with a standard k-type thermocouple with a range of −200 to 1250 ${}^{\circ }$C. Measurement using the lowest speed and highest temperature could not be performed. Ten replicas of the possible combinations of factors were performed to provide a total sample of 80 measurements.

The averages tended to graphically smooth the value of the peak for each replicate. The variability in the measurements was very high. Therefore, the average, considering the frequency values at the peak for each replica with their respective deviations, was considered. The levels of the experimental measurements, including the averages and deviations, are shown in Table 2.

**Figure 2:**
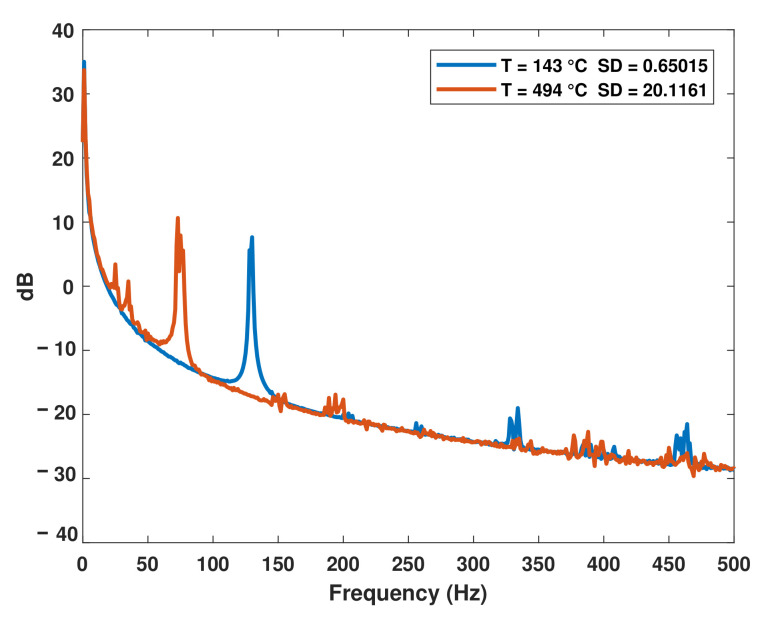
Average measurement of amplitude for runs 5 and 6.

**Figure 3:**
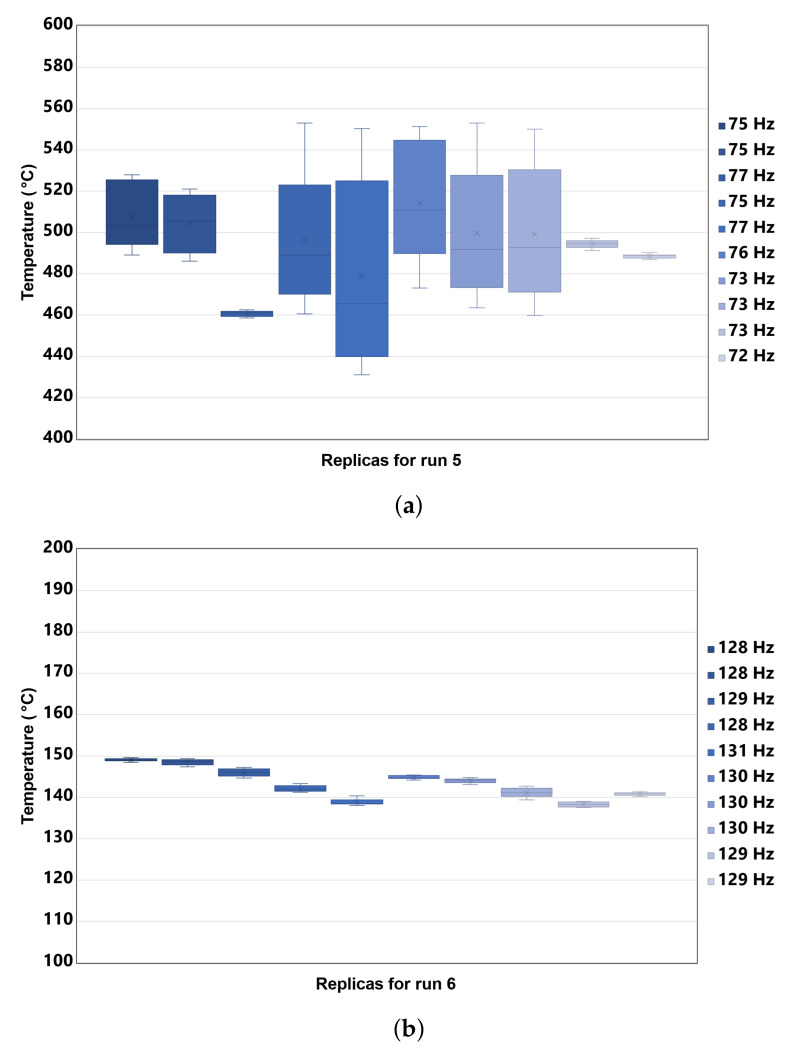
Measurements for runs 5 and 6: (**a**) Replicas of the nominal rotational frequency of 76 Hz with their temperature variability at the nominal temperature of 500 ${}^{\circ }$C; (**b**) Replicas of the nominal rotational frequency of 127 Hz with their temperature variability at the nominal temperature of 150 ${}^{\circ }$C.

Measurements of amplitude versus frequency at nominal temperatures of 150 and 500 ${}^{\circ }$C, corresponding to runs 5 and 6, are presented in Figure 2. The frequency replicas taken for runs 5 and 6 are illustrated in Figure 3. The variability of the frequency replicas and the dispersion of the temperature samples during each amplitude measurement was recorded.

### 2.2. Response Surface Model Tuned by a Metaheuristic Algorithm

Metaheuristic algorithms stand out for their flexibility. However, although the most widely used algorithm is the genetic algorithm (GA), it has some well-known drawbacks. The biggest of these is that it presents a high number of specific parameters. This means that the performance of the algorithm varies considerably with these parameters and there is no standard method to determine them. Jaya, by contrast, has the clear advantage of being an algorithm without specific search parameters. In addition, Jaya is among the simplest algorithms basing its search on the best solutions and moving away from the worst solutions.

**Figure 4:**
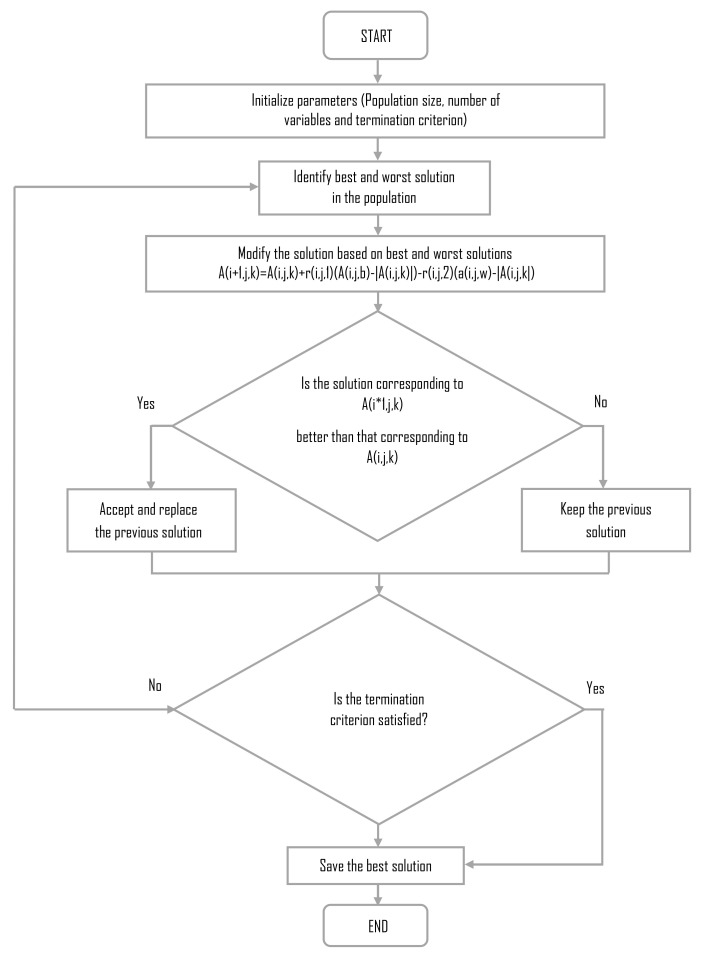
The original Jaya algorithm flow diagram.

There are multiple metaheuristic algorithms without specific parameters. However, according to the “No Free Lunch” theorem, there is no algorithm which is superior to any other if they are averaged in all cases [23]. This is why any metaheuristic algorithm without specific parameters can solve the task. Nevertheless, as previously stated, the Jaya algorithm is among the simplest [24]. The original diagram of the Jaya algorithm is shown in Figure 4, and a full description can be found in [24].

A system based on polynomials is proposed to select the surface response model for estimating the amplitude of vibration as a function of speed and temperature. The amplitude is a function of two polynomials of unknown order and coefficients, as shown in Equation (1). Polynomial models have demonstrated advantages in multiple areas of engineering [25,26]. In this study, a polynomial model was identified and the degree of the polynomial was selected based on performance. However, there is a wide variety of non-linear models that can be tested in future research, such as those analyzed in [27], where the use of logarithmic, exponential, polynomial and trigonometric models is discussed.

(1) $$a_{0}f^{n}+a_{1}f^{n-1}+...+a_{n}f^{0}+b_{0}T^{n}+b_{1}T^{n-1}+...+b_{n}T^{0}=H$$

where *f* is the synchronous rotational frequency of the rotor in Hz, *T* is the temperature in degrees Celsius, and *H* is the amplitude of vibration in μm.

Considering Equation (1), we must find the order *n* of the polynomial and the coefficients *a* and *b* that minimize the error. For this, the general parameters that use the Jaya algorithm are defined. These parameters are shown in Table 3.

In addition to the above parameters, the fitness/cost function must be defined. This is of the utmost importance since it determines how the performance of the model is measured. The algorithm was applied to the eight points which correspond to the average measurements of the levels, as shown in Table 2, and subsequently to the total sample of 80 measurements. The mean square error (MSE) was defined in the 8-point and 80-point runs, as shown in Equation (2). The MSE was used as a performance measure. The solutions with lower RMSE had a higher probability of hatching. A solution with an RMSE of 0 implies a prediction equal to the actual value. The MSE magnifies errors of greater magnitude but reduces those of lower magnitude. The errors expected in the estimates and the experimental measurements are errors of low magnitude. Thus, the MSE is an appropriate indicator for evaluating the performance of the algorithm.

(2) $$MSE=\frac{1}{N}\sum_{i=1}^{N}{\left(x_{i}-{\tilde{x}}_{i}\right)}^{2}$$

where ${\tilde{x}}_{i}$ is the measured point, $x_{i}$ is the estimated point, and *N* is the number of measurement points considered. Therefore, the function with the best fitness is the function with the lowest MSE.

Once the search parameters and the cost function have been defined, it is necessary to find the order of the polynomial that offers the best performance in terms of MSE. For this, multiple tests were carried out with a second-order polynomial. Then the order was gradually increased until it was observed that the increase in the proposed polynomial’s degree did not improve the performance of the algorithm. Ten runs for each order were carried out; the results of these tests are shown in Table 4.

The fifth-order polynomial presented a balance between the complexity and performance according to the previous Table 4. The Jaya algorithm was executed to search the coefficients of a fifth-order polynomial model with normalized magnitudes. Therefore, the final result for the surface response model is described in Equation (3).

(3) $$H\left(f,T\right)=a_{0}f^{5}+a_{1}f^{4}+a_{2}f^{3}+a_{3}f^{2}+a_{4}f+a_{5}+b_{0}T^{5}+b_{1}T^{4}+b_{2}T^{3}+b_{3}T^{2}+b_{4}T+b_{5}$$

## 3. Results

The deviations in temperature up to 27 ${}^{\circ }$C are shown in Table 2; these were most marked at high temperatures. Moreover, the highest rotational frequencies produced the largest deviations (up to 6.44 Hz). The vibration amplitude deviations were more similar, but the largest of 2.1988 μm appeared in the run with the highest temperature and the second rotational frequency.

**Figure 5:**
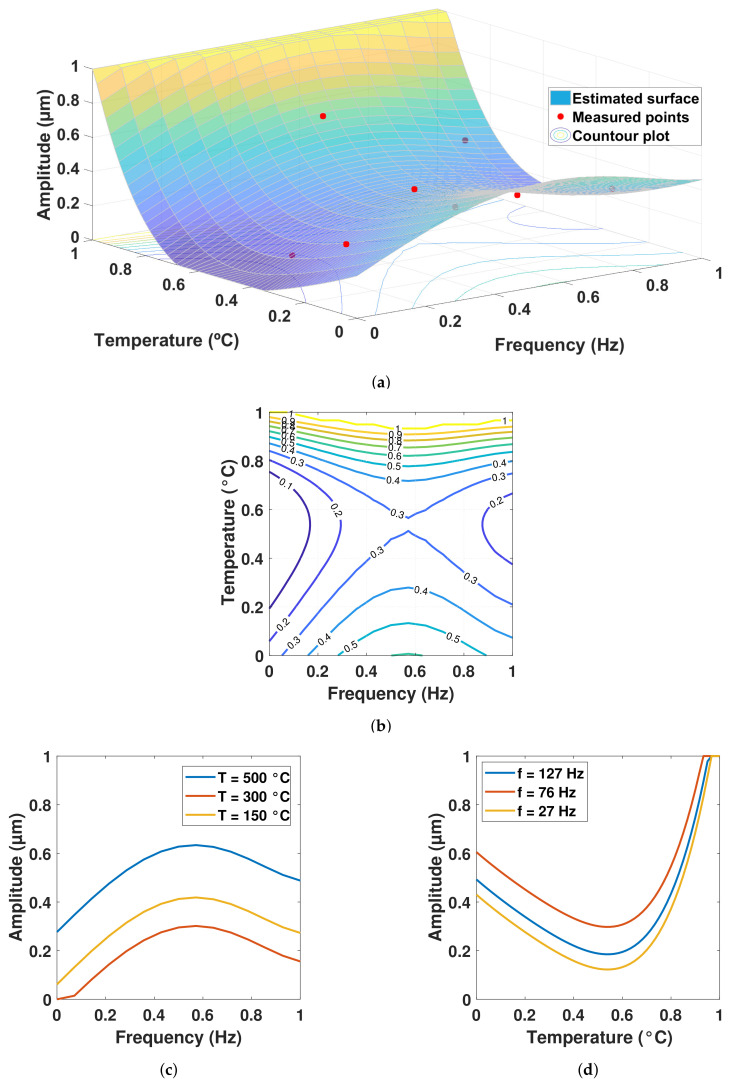
Results from the 5th order polynomial using the 8-point sample: (**a**) Surface response model fitting the sample; (**b**) Contour plot from the surface response model; (**c**) Amplitude vs. frequency when setting the nominal temperature values; (**d**) Amplitude vs. temperature when setting the nominal frequencies.

**Figure 6:**
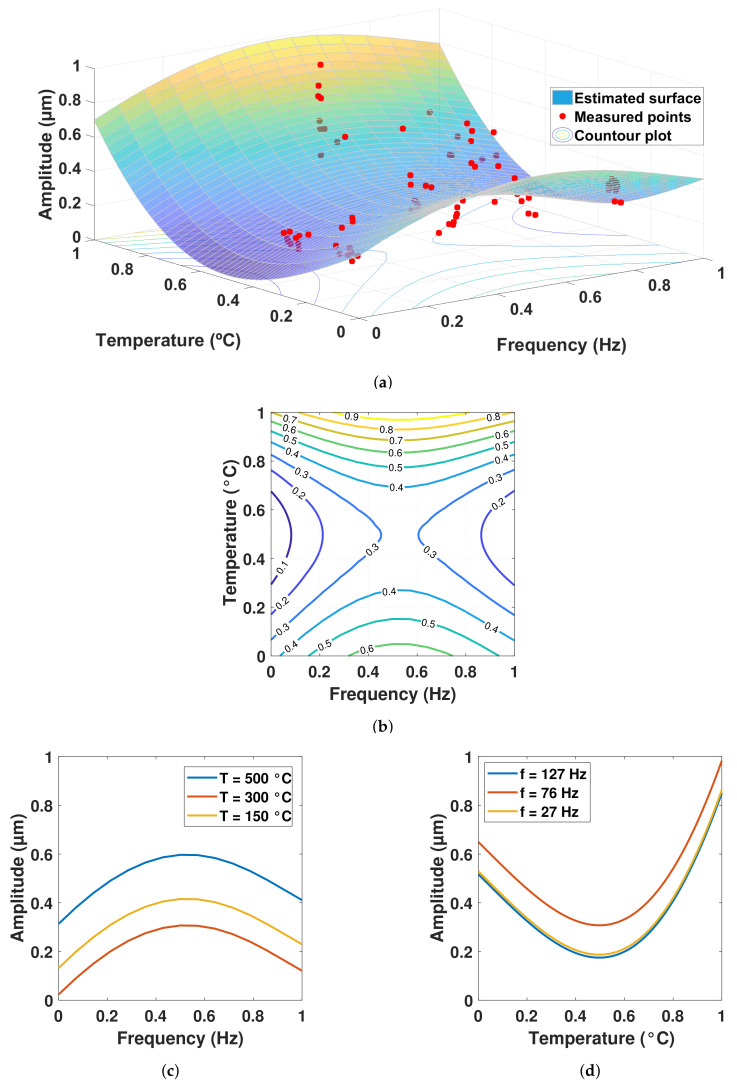
Results from the 5th order polynomial using the 80-point sample: (**a**) Surface response model fitting the sample; (**b**) Contour plot from the surface response model; (**c**) Amplitude vs. frequency when setting the nominal temperature values; (**d**) Amplitude vs. temperature when setting the nominal frequencies.

The coefficients identified by the Jaya algorithm are shown in Table 5 and Table 6 for the 8-point sample, which uses the average measurements, and for the 80-point sample, respectively. The coefficients expressed in “*a*” are those associated with the rotational frequency variable and those of “*b*” with temperature; as can be seen, they are all in a range from −1 to 1. The resulting surface response models using these coefficients from the fifth-order polynomial for both 8-point and 80-point samples are shown in Figure 5 and Figure 6, respectively. The root of the MSE obtained using the 8-point model was 3.12%, while in the 80-point model it was 10.69%.

The contour plots, which were generated from slices of the surface for specific values of vibration amplitude, are found in Figure 5b and Figure 6b. In Figure 5b, the normalized amplitudes of 1 (13 μm) at the top can be seen, while in Figure 6b, they only go up to 0.9 (11.7 μm). The purple outlines in both graphs correspond to the smallest values of the vibration amplitude 1.3–2.6 μm. The contour plots Figure 5b and Figure 6b have similar line distributions, but have areas where they differ at the limits of the vibration amplitude values. This may be because the 8-point model does not take dispersions into account and overestimates vibrations at the highest temperature for the entire frequency range.

Figure 5c and Figure 6c show the response of the vibration amplitude with respect to the entire rotational frequency bandwidth by fixing the nominal temperature values. Figure 5d and Figure 6d show the response of the vibration amplitude with respect to the entire temperature band width, fixing the nominal rotational frequency values.

It is clear that, although both models show approximately the same trend, some differences arise, which are discussed in the next section, where the effects of temperature on the vibration amplitudes and the advantages of the model for design are highlighted.

## 4. Discussion

On the surface model of Figure 5a, which corresponds to the 8-point sample, the smallest values of vibration amplitude are observed for the temperature at a medium level and the rotational frequencies at low and high levels. The steepest rise occurred at medium frequency levels and high temperatures. Although the amplitude also grew at low-temperature levels, the rate of change was not as abrupt as at high temperatures. This behavior can also be seen in Figure 6a for the 80-point sample.

One of the experimental runs that could not be performed would have involved a rotational frequency of 27 Hz and a temperature of 500 ${}^{\circ }$C. Therefore, using the response surface model for both samples, 8-point and 80-point, the vibration amplitude was predicted to be about 5.2 μm. However, if the variation in run 5 is taken to represent the variability of the measurement to be expected, then the amplitude could be between 5.2–6.5 μm.

In Figure 3a, deviations from 1 to 45 ${}^{\circ }$C can be seen, in contrast to Figure 3b, where deviations of up to 1 ${}^{\circ }$C are present. This is because, in runs with lower speeds, it becomes more difficult to reach the higher temperatures. Therefore more gas is required and controlling temperature stabilization is more difficult. Although run 5 had a higher level of temperature compared to run 8, there were also runs with different temperatures that had average deviations very close to 20 ${}^{\circ }$C, reflecting the fact that speed plays an important role since it can facilitate or hinder the air/fuel mixture.

The trajectories in Figure 5c and Figure 6c were very similar to those observed in previous studies [27,28] in which the effect of temperature was not considered. In [27] the gray wolf optimizer (GWO) was used to fit non-linear regression models to the measurements. By including the effect of combustion and high temperatures in this investigation, much more variability was found in the results, and the vibration amplitude levels were up to 45% higher with maximum peaks of 12.06 μm in relation to 6.62 μm for the study referred to. Both maximum peaks were found at the second rotational frequency, which suggests that, at the second level of nominal rotational frequency (76 Hz), a resonance could manifest itself, and no matter the temperature, the vibration will be the highest in this condition. According to [27,28], the deviation in the vibration amplitudes when the effects of temperature were not considered were from 0.2 to 0.7 μm, and when temperature was included, they were from 0.44 to 2.19 μm (see Table 2), which represented a 122.74% increase in the deviation for the mentioned low values, and a 212.85% increase in the deviation for the mentioned high values. The minimum vibration amplitude value of 1.34 μm remained practically the same as in the previous study with a difference of only 2.33%, but it did not appear at the same nominal rotational frequency, changing from the lowest (27 Hz) to the highest (127 Hz), which was attributed to the effect of temperature.

Runs 7 and 8 presented the largest deviations in frequency, as shown in Table 2, requiring more fuel that was more difficult to control, resulting in accelerations and decelerations in the rotational frequency. In terms of amplitude, run 5 presented the maximum value, as well as the maximum deviation. Considering the amplitude results, arguably, the nominal frequency/temperature combinations of 27/300 and 127/150 performed better. Taking this information into account, the turbine appears to be designed to work at an optimum temperature of approximately 300 ${}^{\circ }$C; this would be the appropriate condition thermally to prevent the compressor and its diffuser from suffering damage, as they are made of plastic material. The proposed model could help to determine the most energetically efficient working conditions since the vibration amplitudes at this temperature (300 ${}^{\circ }$C) were the smallest regardless of the frequency range.

The mean absolute percentage difference in Figure 5c was 65.71% between temperatures of 500 ${}^{\circ }$C and 300 ${}^{\circ }$C, which represents an average of 4.27 μm across the entire frequency bandwidth. Comparing the temperatures of 500 ${}^{\circ }$C and 150 ${}^{\circ }$C, the mean absolute percentage difference was 43.81%, equivalent to an average of 1.47 μm. The mean absolute percentage difference in Figure 5d was 39.40% between the frequencies of 76 Hz and 27 Hz, which represents an average of 2.14 μm across the entire frequency bandwidth. Comparing the 76 Hz and 127 Hz frequencies, it was 25.28%, equivalent to an average of 1.37 μm. The mean absolute percentage difference in Figure 6c was 59.71% between temperatures of 500 ${}^{\circ }$C and 300 ${}^{\circ }$C, which represents an average of 3.77 μm across the entire frequency bandwidth. Comparing temperatures of 500 ${}^{\circ }$C and 150 ${}^{\circ }$C, it was 37.42%, equivalent to an average of 1.41 μm. The mean absolute percentage difference in Figure 6d was 27.47% between the frequencies of 76 Hz and 27 Hz, which represents an average of 1.56 μm across the entire frequency bandwidth. Comparing the 76 Hz and 127 Hz frequencies, it was 30.23%, equivalent to an average of 1.72 μm.

The vibration amplitudes in Figure 5c and Figure 6c were similar to those shown in [29], where the amplitudes in the bearings varied with respect to the lubricant supply flow rate of the rotary system. In addition, a “beat vibration” effect was observed by varying the rotational frequency, as in [30]. When the temperature values were fixed, both models (8-point and 80-point) followed a pattern similar to that observed in [31], where the effect of the spindle speed on the amplitude of vibration was significant in any direction and the pattern appeared to be quadratic. It is important to monitor the vibration amplitude with respect to rotational speed, since it has been reported that the former increases with increase in the latter when considering a cracked rotor system [32].

It has been shown that response surfaces enable finding of the optimal conditions for a vibration amplitude [33], and, in our case, this happened when using 27 Hz and 300 ${}^{\circ }$C with a predicted amplitude of 2.1619 μm against a real value of 1.3442 μm. As mentioned above, higher frequency values at the same temperature produce lower values of amplitude.

In [34], a response surface methodology and ANOVA were used to study the effect of various parameters on the vibration amplitudes and tool wear in a turning process. The authors obtained experimental coefficients of determination up to 98%. When the 8-point model was used in our investigation, the coefficient of determination was 95.40%, while, with the 80-point model, the coefficient of determination fell to 58.20%. However, the error remained close to 10%. The same procedure was used to find the geometric and machining parameters that would minimize the vibration amplitude in Al7075-T6 aluminum milling processes [35]. As previously stated, the temperature effect produced higher vibration amplitudes, and the minimum amplitude value appeared at different rotational frequencies. Therefore, it is important to consider this analysis in terms of design. However, the disadvantage of having so much variability in the experimental data, especially in temperature, is that the fit of the regression model is reduced.

Artificial intelligence algorithms, such as the artificial neural network (ANN), have been shown to produce better response models than the response surface method [36]. Artificial intelligence, with an adaptive-network-based fuzzy inference system (ANFIS), has been used to optimize an ultrasonic vibration-assisted sheet-metal-forming process [37]. Response surfaces were obtained where the interactions of the vibration amplitude with the forming force and the feed rate were evaluated. The estimation errors with respect to the experiments reached 10%, which was very close to the 10.69% estimation error of the 80-point model.

The proposed response surfaces can be used to identify trajectories that allow the establishment of operating modes, for example, vibrations versus fuel consumption, vibrations versus work speed, or the establishment of a maintenance plan. They can also help to identify vibration modes in a modal analysis where bending modes are not easy to detect in the rotor frequency response functions.

## 5. Conclusions

Vibration amplitude measurements were obtained by varying the rotational frequency and the temperature in a gas microturbine. Two response surface models were constructed with a metaheuristic algorithm applied to a sample using the averages of the experimental levels (8-point) and a sample with all observations (80-points). It was shown that the model with the averages predicted the same trends as the model with the whole sample. Although the estimated error using all the observations (80-point) was higher, the model that used the averages of the experimental levels could be used when a lower degree of variability is guaranteed in the experiments.

The best operating conditions were found at low and high rotational frequencies and at a temperature of 300 ${}^{\circ }$C. The high temperature conditions produced more variability in the measurements. The effect of temperature caused the minimum vibration amplitude value to not appear at the same rotational frequency as in the previous study where temperature was not considered, changing from the lowest (27 Hz) to the highest (127 Hz).

The methodology implemented in this study can be used to determine the vibration response under difficult operating conditions to achieve, for example, combinations of low rotational frequencies and high temperatures. This research represents an innovative approach for the estimation of response surfaces that fit measurements with high variability, not only of vibration, by employing metaheuristic algorithms. The proposed response surfaces highlight paths that enable the establishing of operating modes, the development of maintenance plans, or of monitoring systems that could send an alert when the measurement points have a surface deviation from pre-established values recorded in standard or historical files.

Future research will investigate the use of different error functions which cannot be conveniently implemented in a common second-order model. The proposed fifth-order model could be replaced by another function different from the polynomial, which may increase the computational cost depending on the non-linearity of the model. If the computational cost is not relevant, the algorithm can be implemented with non-linear models and the fit can be evaluated when the data contains significant variability.

## Figures and Tables

**Table 1:** Technical data for the microturbine.

Parameter	Description
Fuel	Butane/propane gas with maximum pressure of 3.5 kg/cm${}^{2}$
Turbine blades outer/inner diameter	68.6/40.5 mm
Compressor wheel outer/inner diameter	64.5/32.8 mm
Turbine wheel diameter	70 mm
Burner hole spacing	10 mm
Number of gas outlet holes	16

**Table 2:** Average levels of the experimental runs.

Run	Temperature (${}^{\circ }$C)	Standard Deviation	Frequency (Hz)	Standard Deviation	Amplitude (µm)	Standard Deviation
1	151	17.8558	22.7	1.9465	3.4074	1.2252
2	291	5.0394	25.7	1.0593	2.3028	0.4475
3	145	0.4883	65.9	4.2804	4.8497	1.7376
4	302	2.5995	77.3	2.4517	4.2910	1.8773
5	494	20.1161	74.6	1.7763	8.0382	2.1988
6	143	0.6501	129.2	1.0328	4.2209	0.6005
7	298	23.3357	118.4	6.3805	2.5392	1.2526
8	468	27.1826	127.2	6.4472	4.7098	1.1205

**Table 3:** General parameters used by the Jaya algorithm.

Parameter	Value	Description
Population	5000	Number of vectors of proposed solutions
Variables	12	Length of coefficient vector
Maximum generations	5000	Maximum number of iterations
Low boundary	−1	Lower limit of search
Up boundary	1	Upper limit of search

**Table 4:** Fitting error varying the order of the polynomial.

Order	Root of Average MSE (%)
2	7.3524
3	4.7830
4	3.5040
5	3.4413
6	3.7629

**Table 5:** Coefficients obtained for a 5th order polynomial model by means of the Jaya algorithm using the 8-point sample.

Coefficient	Value	Coefficient	Value
$a_{0}$	1	$b_{0}$	1
$a_{1}$	−1	$b_{1}$	1
$a_{2}$	−0.6365	$b_{2}$	−1
$a_{3}$	−0.1521	$b_{3}$	0.6398
$a_{4}$	1	$b_{4}$	−0.8662
$a_{5}$	−0.7513	$b_{5}$	1

**Table 6:** Coefficients obtained for a 5th order polynomial model by means of the Jaya algorithm using the 80-point sample.

Coefficient	Value	Coefficient	Value
$a_{0}$	0.3677	$b_{0}$	−0.6255
$a_{1}$	−0.2627	$b_{1}$	1
$a_{2}$	−0.3477	$b_{2}$	1
$a_{3}$	−0.6472	$b_{3}$	−0.0410
$a_{4}$	0.9872	$b_{4}$	−1
$a_{5}$	−0.6344	$b_{5}$	1

## Data Availability

The data presented in this study are available on request from the corresponding author.
